# Bibliometric analysis of publications on genetic polymorphism and external apical root resorption research

**DOI:** 10.1590/2177-6709.29.4.e2423268.oar

**Published:** 2024-09-02

**Authors:** Liz Helena Moraes PINHEIRO, Dalila Ferreira Silvano de MOURA, Leonardo Santos ANTUNES, Lívia Azeredo Alves ANTUNES

**Affiliations:** 1 Fluminense Federal University, Postgraduate Program in Dentistry (Niterói/RJ, Brazil).; 2 Fluminense Federal University, Nova Friburgo Institute of Health, School of Biomedicine (Nova Friburgo/RJ, Brazil).; 3 Fluminense Federal University, Nova Friburgo Institute of Health, Department of Specific Formation (Nova Friburgo/RJ, Brazil).

**Keywords:** Genetic polymorphism, Root resorption, External apical root resorption, Bibliometrics, Polimorfismo genético, Reabsorção radicular, Reabsorção radicular apical externa, Bibliometria

## Abstract

**Objective:**

This study aimed to analyze the scientific production of genetic polymorphisms and external apical root resorption (EARR) to establish main findings, geographic trends, and research gaps for possible future investigations.

**Methods:**

Unrestricted publications were searched using the Scopus database (March 2023) to include studies that addressed the association between genetic polymorphisms and EARR. Case-control, cohort, cross-sectional, and review studies were considered eligible. The softwares VOS viewer™ and Bibliometrix were used for data analysis.

**Results:**

Of the 44 studies analyzed, “Iglesias-Linares A” was the most cited author. The University of Seville (Spain) conducted the most research on this topic. Brazil, Spain, and the USA were the leading countries in terms of citations. The most frequent term in the co-occurrence of keywords was “EARR.” The journal American Journal of Orthodontics and Dentofacial Orthopedics presented a great relevance in the area, demonstrating a high number of publications. Several genetic polymorphisms have been investigated, with interleukins being the most studied.

**Conclusion:**

Endodontics is an area of research that should focus more on root resorption and genetic polymorphisms, as it still underexplored, compared to orthodontics. Polymorphisms have been studied as possible predictors of EARR caused by orthodontic tooth movement. However, the gap in the research indicates a need to search for new genes associated with EARR.

## RESUMO

**Objetivo:**

Este estudo objetivou analisar a produção científica sobre polimorfismo genético e reabsorção radicular apical externa (external apical root resorption, EARR) para estabelecer os principais achados, tendências geográficas e lacunas de pesquisa, para possíveis investigações futuras.

**Métodos:**

Foram pesquisadas publicações na base de dados Scopus (março de 2023) para incluir estudos que abordassem a associação entre polimorfismos genéticos e EARR. Foram considerados elegíveis estudos de caso-controle, relatos de caso, coorte, transversais e revisões. Para análise dos dados, foram utilizados os softwares VOS viewer® e Bibliometrix.

**Resultados:**

Dos 44 estudos incluídos, Iglesias-Linares A foi o autor mais citado. A Universidade de Sevilha (Espanha) foi a instituição que mais pesquisou o tema. Brasil, Espanha e EUA foram os principais países em termos de citações. O termo mais frequente na coocorrência das palavras-chave foi EARR. O periódico American Journal of Orthodontics and Dentofacial Orthopedics apresentou grande relevância na área, com vários artigos publicados. Vários polimorfismos genéticos foram investigados, sendo as interleucinas as mais estudadas.

**Conclusão:**

Endodontia é uma área de pesquisa que poderia focar mais no tema reabsorção radicular e polimorfismos genéticos, por ser um assunto ainda pouco explorado, em comparação à Ortodontia. Polimorfismos têm sido estudados como possíveis genes preditores de EARR como consequência da movimentação dentária ortodôntica. No entanto, ainda existe uma lacuna na investigação, indicando a necessidade de procurar novos genes associados à EARR.

## INTRODUCTION

Root resorption is a local physiological process, when it occurs in healthy deciduous teeth; or pathological, when it occurs in damaged permanent and deciduous teeth. Pathological resorption may be a consequence of many factors, such as: traumatic and infectious factors, orthodontic movement and applied forces, impacted teeth, chronic bruxism, and periapical lesions.^1^

Recognized as a multifactorial process that includes associated genetic factors,^2^ external apical root resorption (EARR) has been investigated by several dental researchers. Two systematic reviews^3^ ^,^ ^4^ showed robust evidence concerning the association between EARR and genetic factors, and other pertinent studies have also demonstrated this association.^2^ ^-^ ^10^

Human genetic diversity is characterized by single nucleotide polymorphisms (SNP).^11^ Genetic polymorphisms are DNA sequence variations;^8^ ^,^ ^12^ currently, individual genomic profiles are evaluated with increasing efficiency, highlighting the pathological predispositions associated with polymorphic genes, such as individual susceptibility to develop EARR due to different stimuli, including orthodontics movement.^2^ ^-^ ^10^ ^,^ ^12^

Expanding the knowledge of EARR is essential for professionals to act coherently and scientifically to diagnose and treat it clinically. Bibliometrics is a quantitative analysis method used in scientific research. The collected data reflect current research trends and help to identify topics for future research for a better understanding of scientific dynamics.^13^

The objective of this bibliometric review was to analyze the scientific production in the field of genetic polymorphisms and EARR to establish findings, geographic trends and research gaps for possible perspectives for future investigations. 

## MATERIAL AND METHODS

All analyzed data were collected from the Scopus database.^14^ The search was conducted in March 2023, with no limitations regarding the language or year of publication.

The following search strategy was used in this review: #1((TITLEABSKEY (genetic AND polymorphism)) AND #2 (TITLEABSKEY (root AND resorption)). The terms “external apical root resorption,” “EARR,” “genes,” and “genetics” were tested in the primary search and no difference in the results was observed (i.e., the number of studies included was not influenced by the addition of these words). Therefore, this search key was selected because it was more sensitive to the proposed theme. 

All detected studies were saved in the software (Zotero 6.0.18). After that, the eligibility criteria was applied. Case-control, cohort, cross-sectional, and review studies that addressed the association between genetic polymorphisms and EARR were included. Case reports, book chapters, *in-vitro* and animal studies, and articles that mentioned only bone resorption and not root resorption were excluded. Based on these criteria, two independent reviewers (LHMP and DFSM) selected articles accessed by title and abstract. Studies with unclear abstracts and titles were read in full, to minimize the possibility of disregarding pertinent studies, and the inclusion and exclusion criteria were applied. Articles that generated disagreements between the reviewers were discussed and reviewed by a third author (LAAA), thus reaching an agreement. 

### BIBLIOMETRIC ANALYSIS

The softwares VOS viewer™ (version 1.6.18) for Mac^15^ and Bibliometrix^16^ were used for visualizing bibliometric analyses. CSV file containing the citation and bibliographic information, abstract, and keywords was used. The VOS viewer™ software was used to analyze the characteristics of the publications, including the most cited researchers, most relevant affiliations, most cited countries, countries with the highest publication production, co-occurrence of keywords, most cited sources, most relevant authors and most cited documents, resulting in maps and graphs for visualization and interpretation of the data obtained from the analyses. The studied items were grouped into clusters, a set of items included in a map, and labeled using cluster numbers. For easier interpretation, the items were called “nodes”, the links presented between them were referred to as “edges”, and the strength of the links was interpreted as the “edge weight”. Depending on the selected analysis, different nodes represented different terms, and their sizes indicated the number of citations, co-citations, and co-occurrences of keywords. The nodes and lines belonging to the same cluster had the same color. A network visualization map of the most cited researchers, network visualization map of the most cited countries, most cited documents, and timeline of the corresponding publications was generated in the VOS viewer™.

Bibliometrix software is an open-source tool that covers a range of quantitative analyses, favoring the visualization of the results. Maps and graphs were generated, and data matrices were constructed for co-citation, scientific collaboration, and keyword analyses.

The synthesis of relevant narrative data (data extraction) was independently done by two reviewers (LHMP and DFSM). It was presented in detail, and grouped by year of publication. The data of the selected articles were allocated and organized in tables: Table 1 - Description of the selected studies about genetic polymorphisms associated to EARR; Table 2 - sample characteristics of the most cited studies in the field of orthodontics; and Table 3 - analyses of revisions included in the bibliometrics.

**Table 1:** Description of the selected studies about genetic polymorphisms associated to EARR.

Authors/Year/ Area of dental research	Genes (SNP) evaluated	Polymorphism Association with EARR	
		Yes	No
*Sharab et al.^2^ (2015) Orthodontics	P2RX7 (rs208294, rs1718119, rs2230912) CASP1/ICE (rs580253, rs554344, rs530537 IL1-B (rs1143634) IL1-A (rs1800587) IL1RA (rs41959)	P2RX7 (rs208294)	P2RX7 (rs1718119, rs2230912) CASP1/ICE (rs580253, rs554344, rs530537 IL1-B (rs1143634) IL1-A (rs1800587) IL1RA (rs41959)
*Al-Qawasmi et al.^5^ (2003) Orthodontics	IL1-A (rs1143634) IL1-B (rs1143627)	IL1-B (rs1143627)	IL1-A (rs1143634)
*Bastos Lages et al.^6^ (2009) Orthodontics	IL-1B (rs1143634)	IL-1B (rs1143634)	______
*Linhartova et al.^7^ (2013) Orthodontics	IL1A (rs16347) IL1B (rs1143627) IL1RN (rs9005)	IL1A (rs16347) IL1B (rs1143627) IL1RN (rs9005)/Short alleles	IL1B (rs1143627) IL1RN (rs9005)/Long alleles
*Iglesias-Linares et al.^8^ (2012) Endodontics	IL1A(rs1800587) IL1B (rs1143634)	IL1B (rs1143634)	IL1 (rs1800587)
*Guo et al.^9^ (2016) Orthodontics	IL-6 (rs1800796) IL1RN (rs419598)	IL-6 (rs1800796)	IL1RN (rs419598)
Borges de Castilhos et al.^10^ (2019) Orthodontics	RANKL (rs1038434, rs3742257, rs931273, rs12585229) RANK (rs12455775) OPG (rs2875845, rs3102724, rs1032128, rs3102728)	RANK (rs12455775) OPG (rs2875845, rs3102724, rs1032128, rs3102728)	RANKL (rs1038434, rs3742257, rs931273, rs12585229)
*Iglesias-Linares et al.^12^ (2012) Orthodontics	IL1A (rs1800587) IL1B (rs1143634) IL1RN (rs419598)	IL1B (rs1143634) IL1RN (rs419598)	IL1A (rs1800587)
*Tomoyasu et al.^17^ (2009) Orthodontics	IL-1B (rs1143634)	______	IL-1B (rs1143634)
*Pereira et al.^18^ (2014) Orthodontics	OPG (rs3102735) RANK (rs1805034) IL-1B (rs1143634) P2RX7 (rs1718119)	P2RX7 (rs1718119)	OPG (rs3102735) RANK (rs1805034) IL-1B (rs1143634)
*Borilova Linhartova et al.^19^ (2017) Orthodontics	IL17A (rs2275913) P2RX7 (rs208294, rs1718119) SSP 1(rs9138, rs11730582) OPG (rs2073618, rs3102735)	P2RX7 (rs208294, rs1718119)	PPS 1 (rs9138, rs11730582) OPG (rs2073618, rs3102735)
*Fontana et al.^20^ (2012) Orthodontics	Vitamin D receptor TaqI	Vitamin D receptor TaqI	______
*Iglesias-Linares et al.^21^ (2014) Orthodontics	OPG (rs9138, rs11730582)	OPG (rs9138, rs11730582)	______
Urban and Mincik^22^ (2010) Endodontics	IL-1A IL-1B IL1RN	IL-1B	IL-1A IL1RN
**Lee et al.^25^ (2022) Orthodontics	SPP1 (rs 4754, rs1126616, rs9138) SFRP2 (rs3810765)	SPP1 (rs9138) SFRP2 (rs3810765)	SPP1 (rs 4754,rs1126616)
*Rutsch et al.^26^ (2015) other areas of dental research	MDA5 IFIH1	MDA5 IFIH1	______
*Al-Qawasmi et al.^27^ (2003) Orthodontics	RANK (rs1805034) TNSALP (rs1256328) TNF-a (rs1799724)	RANK (rs1805034)	TNF-a (rs1799724) TNSALP (rs1256328)
*Gülden et al.^28^ (2009) Orthodontics	IL1-A (-889) IL1-B (+3954) (rs1143634)	IL1-A (-889) IL1-B (+3954)	IL-1B (rs1143634)
*Iglesias-Linares et al.^29^ (2017) Orthodontics	IL1-B (rs1143634) IL1RN (rs419598) SPP1 (rs9138, rs11730582)	IL1RN (rs419598)	IL1B (rs1143634) SPP1 (rs9138, rs11730582)
*Pettersson et al.^30^ (2017) Other areas of dental research	MDA5 IFIH1	MDA5 IFIH1	______
Pereira et al.^31^ (2016) Orthodontics	IL1B (rs1143634) IL1RN (rs315952) IRAK1 (rs1059703)	IRAK1 (rs1059703)	IL1B (rs1143634) IL1RN (rs315952)
Bastos et al.^32^ (2015) Other areas of dental research	IL1A (rs 180058) IL1B (rs1143634) IL1RN (rs419598)	______	IL1A (rs 180058) IL1B (rs1143634) IL1RN (rs419598)
Ciurla et al.^33^ (2021) Orthodontics	IL-1B (rs1143634) TNFRSF11B (rs3102735) CASP1(rs530537) IL-6 (rs1800796)	IL-1B (rs1143634)	TNFRSF11B (rs3102735) CASP1(rs530537) IL-6 (rs1800796)
Roskamp et al.^34^ (2017) Endodontics	IL4 (rs2227284, rs2243268)	______	IL4 (rs2227284, rs2243268)
Roskamp et al.^35^ (2018) Endodontics	IL4 (rs2227284, rs2243268)	______	IL4(rs2227284, rs2243268)
Behnaz et al.^36^ (2020) Orthodontics	IL-1A (rs1800587) IL-1B (rs1143634)	IL-1B (rs1143634)	IL-1A (rs1800587)
Ciurla et al.^37^ (2021) Orthodontics	P2RX7 (rs208294) IL1RN (rs419598)	P2RX7 (rs208294) IL1RN (rs419598)	______
Iber-díaz et al.^38^ (2020) Orthodontics	Multiple putative loci and genes located at somatic chromosomes 2, 4, 8, 12, 18, and at sexual chromosomes X and Y	STAG (rs151184635) RP1-30E17.2 (rs55839915) SSP1 (rs11730582) P2RX7 (rs1718119) TNFRSF11A (rs8086340)	Other loci and genes located on somatic chromosomes 2, 4, 8, 12, 18 and on sex chromosomes X and Y analyzed
Roskamp et al.^39^ (2021) Other areas of dental research	IL-6 (rs2069843)	IL-6 (rs2069843)	______
Bahirrah et al.^40^ (2023) Orthodontics	IL-IB (rs1143634)	______	IL-IB (rs1143634)
Marañón‐Vásquez et al.^41^ (2023) Orthodontics	VDR (rs1544410, rs7975232, rs731236, rs2228570) GC (rs4588) P450CYP27B1 (rs4646536) P450 CYP24A1(rs921650)	VDR (rs2228570) P450 CYP27B1 (rs4646536) P450 CYP24A1- less expressive	VDR (rs1544410, rs7975232, rs731236) GC (rs4588)
Silva et al.^42^ (2022) Orthodontics	TNFRSF11B gene, OPG (rs3102735) TNFRSF11A gene, RANK (rs1805034) IL-IB (rs1143634) P2RX7 (rs1718119) IL1RN (rs315952)	TNFRSF11B gene, OPG (rs3102735) P2RX7 (rs1718119) IL1RN (rs315952)	TNFRSF11A gene, encoding RANK (rs1805034) IL-IB (rs1143634)
Roskamp et al.^43^ (2022) Other areas of dental research	IL4(rs2227284/rs2243268) IL6(rs1524107/rs2069835/rs2069838/rs2069840/rs2069843/rs2069845)	______	IL4(rs2243268)
Baghaei et al.^44^ (2023) Orthodontics	IL-1A (rs1800587) IL-1B (rs1143634) \ IL-1RN (rs419598) P2RX7 (rs1718119, rs2230912 IRAK1 (rs1059703) CASP1 (rs530537, rs580253, rs554344)	IL-1A (rs1800587)	IL-1B (rs1143634) IL-1RN (rs419598) P2RX7 (rs1718119, rs2230912), IRAK1 (rs1059703) CASP1 (rs530537, rs580253, rs554344)
*Iglesias-Linares et al.^47^ (2012) Endodontics and Orthodontics	IL1A(rs1800587) IL1B (rs1143634)	IL1B (rs1143634)	IL1A (rs1800587)
Iglesias-Linares et al.^48^ (2013) Endodontics and Orthodontics	IL1RN (rs419598)	IL1RN (rs419598)	_____
*Iglesias-Linares et al.^49^ (2016) Other areas of dental research	IL-1B (rs1143634) IL1A (rs1800587) IL1RN (rs419598) IL-6 (rs1800796) P2RX7 (rs208294, rs1718119, rs2230912) TNFRSF11B (OPG) TNFRSF11A (rs3102735, rs1805034) RANKL (rs228769)	IL-1B (rs1143634) IL1A (rs1800587) P2RX7 (rs208294) TNFRSF11B (OPG) TNFRSF11A (rs3102735, rs1805034) RANKL (rs228769)	IL1RN (rs419598) P2RX7 (rs1718119, rs2230912)

EARR = external apical root resorption. IL-1A (-B, -RN, -4, -6) = interleukin α (-β, -receptor antagonist). P2XR7 = purinoreceptor P2X7. SPP1 = secreted phosphoprotein 1 or osteopontin. TNF- α = tumor necrosis factor α. RANK = Receptor activator of nuclear factor-κB RANK. TNFRSF11B = Tumour Necrosis Factor Receptor Superfamily Member 11a gene, encoding receptor activator of nuclear factor-κB RANK. TNFRSF11B = tumor necrosis factor receptor superfamily member 11B. TNSALP = tissue non-specific alkaline phosphatase. IRAK1 = Interleukin 1 Receptor Associated Kinase. CASP1/ICE = caspase-1/interleukin-converting enzyme. IL1RA = a interleukin-1 receptor antagonist. MDA5 = Melanoma Differentiation-Associated protein 5. IFIH1 = interferon induced with helicase C domain 1. IL1RN = interleukin 1 receptor antagonist gene. OPG = osteopontin gene. VDR = vitamin D receptor. CYP27B = cytochrome P450 family 27 subfamily B member 1. CYP24A1 = cytochrome P450 family 24 subfamily A member 1. STAG2 = stromalantigen2. RP1-30E17.2 = Clone-based(Vega)gene. SPP1 = osteopontin. *Most cited article, according to Figure 6.

**Table 2:** Sample characteristics of the most cited studies of the Orthodontics area.

Author/ year	Population	Sample (n) and study design	Age in years Mean (SD)	Orthodontic technique	Methods used to detect EARR	Imaging exams	Evaluated tooth/teeth	Angle malocclusion
Al-Qawasmi et al.^5^ (2003)	USA	n=118 (73 siblings and 45 parents) / Pretreatment and postreatment	12.1 (1.89)	Full-banded comprehensive treatment	Harris et al. (1997)	Lateral cephalometric Panoramic radiographs	Maxillary central incisor, Mandibular central incisor, Mandibular first molar, mesial root and Mandibular first molar, distal root	Class I Class II Class III
Al-Qawasmi et al.^27^ (2003)	USA	n=124 (79 siblings and 45 parents)	12.3 (1.82)	Full-banded comprehensive treatment	Harris et al. (1997)	Lateral cephalometric Panoramic radiographs	Maxillary central incisor, Mandibular central incisor, Mandibular first molar, mesial root and Mandibular first molar, distal root	Class I Class II Class III
Bastos Lages et al.^6^ (2009)	Brazil	n = 61 EARR = 23 Control = 38	18.9 (5.2)	Straight wire	Linge and Linge’s method (1991) modified by Brezniak (2004)	Periapical radiographs	Maxillary incisors (central and lateral)	Class I Class II Class III
Sharab et al.^2^ (2015)	USA	n = 134 EARR = 67 Control = 67	EARR: 15.78 (1.13) Control: 15.79 (1.14)	Edgewise	Malmgren et al. (1982)	Panoramic radiographs Occlusal radiographs	Maxillary incisors (central and lateral)	NR
Gülden et al.^28^ (2009)	Germany	n = 258 EARR = 96 Control = 162	NR	NR	Linge and Linge’s method (1983)	Panoramic radiographs scanned	Canines, premolars and molars (mesial and distal roots)	NR
Iglesias-Linares et al.^29^ (2017)	Spain	n=372 EARR = 174 Control = 198	NR	Removable aligners Straight wire	Harris et al. (1997) Al-Qawasmi et al. (2003)	Panoramic radiographs	Maxillary incisors (central and lateral)	Class I Class II Class III
Iglesias-Linares et al.^47^ (2013)	Spain	n = 93 EARR = 39 Control = 54	EARR: 24.54 (5.85) Control: 23.89 (5.72)	Straight wire	According to Linge and Linge’s method (1991) modified by Brezniak (2004)	Lateral cephalometric Panoramic radiographs	Upper second root-filled premolars	Class I Class II Class III
Fontana et al.^20^ (2012)	Brazil	n = 377 EARR = 339 Control = 38	14.9 (NR)	Edgewise or Straight wire techniques	According to Linge and Linge’s method (1991)	Periapical radiographs	Maxillary central incisors	Class II, division 1
Iglesias-Linares et al.^12^ (2012)	Spain	n = 73 EARR = 30 Control = 43	23.78 (5.91)	Straight Wire	Linge and Linge (1991) modified by Brezniak (2004)	Lateral cephalometric Panoramic radiographs	Upper premolars (vital and rooted teeth. Split-mouth and parallel group design)	Class I Class II Class III
Pereira et al.^18^ (2014)	Portugal	n = 195 Pretreatment and postreatment	17.24 (6.8)	Hyrax and Straight wire	Linge and Linge’s method (1991) modified by Brezniak (2004)	Lateral cephalometric Panoramic radiographs	Maxillary incisors (central, lateral) Maxillary canines	Class I Class II Class III
Linhartova et al.^7^ (2013)	Czech Republic	n = 106 EARR = 32 Control = 74	15.2 (5.0)	Straight wire or segmental technique	Linge and Linge’s method (1991) modified by Brezniak (2004)	Lateral cephalometric Panoramic radiographs	Maxillary incisors (central and lateral)	Class I Class II Class III
Guo et al.^9^ (2016)	Han Chinese	n = 174 Pretreatment and postreatment	14.07 (3.1)	Straight wire	NR	Cone beam computed tomography	Maxillary incisors (left central)	Class I Class II Class III
Iglesias-Linares et al.^21^ (2014)	Spain	n = 87 EARR = 37 Control = 50	EARR: 24.7 (5.95) Control: 23.8 (5.33)	Straight wire	Linge and Linge’s method (1991) modified by Brezniak (2004)	Lateral cephalometric Panoramic radiographs	Maxillary incisors (central, lateral)	Class I Class II Class III
Tomoyasu et al.^17^ (2009)	Japan	n = 54 Case (EARR > 2.0 mm) = NR Control (EARR < 2.0 mm) = NR	NR	NR	Harris et al. (1997)	Lateral cephalometric Panoramic radiographs	Maxillary and mandibular central incisors Mandibular first molar mesial root and distal root	Class I Class II Class III
*Borilova Linhartova et al.^19^ (2017)	Czech Republic	n = 99 EARR = 30 Control = 69	EARR: 14.6 (3.2) Control: 15.2 (5.3)	Fixed orthodontic appliance therapy	Linge and Linge’s method (1991) modified by Brezniak (2004)	Lateral cephalometric Panoramic radiographs	Maxillary incisors (central, lateral)	Class I Class II Class III

NR = not reported, EARR = external apical root resorption.

*Most cited article in orthodontics, according to Figure 6.

**Table 3:** Analysis of revisions included in the bibliometrics.

Authors/Year	Study Type	Conclusion
Pinheiro et al.^3^ (2021)	Systematic review and meta-analyses	Narrative analyses of individual studies demonstrated an association of many genes. The number of studies for each genetic variation was very low, and methodological heterogeneity between the studies was observed. The meta-analysis could only show an involvement for P2RX7 (rs208294) in the risk of orthodontic patients to EARR at a very low certainty of evidence according to GRADE
*Nowrin et al.^4^ (2018)	Systematic review and meta-analyses	More research on the relationship between gene polymorphism and EARR is necessary to determine better specificity of possible interactions.
Wu et al.^23^ (2013)	Meta-analyses	The variant genotypes (CC and CT) on polymorphism to IL-1B were not associated with EARR risk, compared with the wild-type TT homozygote
Aminoshariae et al.^24^ (2016)	Systematic review	The current investigation suggests guidelines and recommendations for future investigators studying genetic polymorphism in patients undergoing orthodontic treatment
Nieto-Nieto et al.^45^ (2017)	Literature review	In recent years, international research groups have determined the degree of influence of some genetic biomarkers in the definition of increased/reduced susceptibility to post-orthodontic EARR. The influences of the gene cluster IL1 (IL1B, IL1A, IL1RN, IL6), P2RX7, CASP1, OPG (TNFRSF11B), RANK (TNFRSF11A), Osteopontin (OPN), TNFa, the vitamin D receptor (TaqI), TNSALP and IRAK1 were the most analyzed
Pereira et al.^46^ (2018)	Literature review	This study was performed searching for the association of rs1800587 from Interleukin-1 alpha (IL1A) gene and rs1143634 from interleukin-1 beta (IL1B) gene with EARR. In conclusion, suggests that for IL1B SNP rs1143634 and EARR have an opposite genetic profile. For IL1A, the hypothesis could not be confirmed
Behnaz et al.^50^ (2020)	Concise review	The rs1800587 and rs1143634 in IL-1A and IL-1B genes have been the mostly assessed SNPs in different populations. Yet, the results of investigations in different populations are not consistent. In this study, the authors summarize the results of studies that assessed the contribution of genetic factors in EARR. As genetic factors are involved in conferring risk of EARR, evaluation of these variants prior to establishment of orthodontic treatments might help in identification of at-risk individuals and better follow-up of these patients

EARR = external apical root resorption. IL-1 (-A, -B, -RN, -6) = interleukin. P2XR7 = P2X7 purine receptor. TNF- α = tumor necrosis factor α. TNFRSF11A = tumor necrosis factor receptor superfamily member 11A, RANK. TNFRSF11B = tumor necrosis factor receptor superfamily member 11B. OPG = osteopontin gene. TNSALP = tissue non-specific alkaline phosphatase. IRAK1 = receptor-associated kinase interleukin 1. CASP1/ICE = caspase-1/interleukin-converting enzyme.

GRADE = Grading of Recommendations Assessment, Development, and Evaluation working group.

* Description of the most cited studies according to Figure 6.

## RESULTS

**Figure 1:**
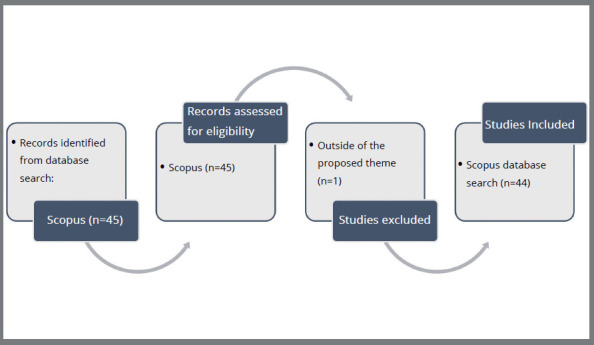
Flow diagram of the selection process.

A search was conducted, and 45 articles were retrieved from the Scopus database using the search strategy. Considering the eligibility criteria, one article was unrelated to the theme. Finally, 44 publications were retrieved and included in the bibliometric analysis (Fig 1).

### MAIN COLLABORATION AND CO-AUTHORSHIP NETWORKS

**Figure 2:**
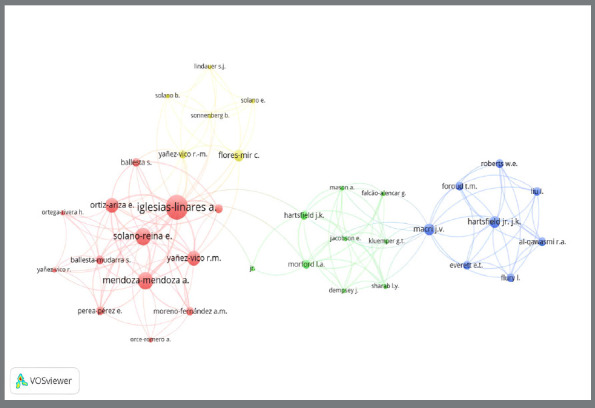
Network visualization map of the most cited researchers.

Concerning the author’s impact on the subject, approximately 200 researchers among the 44 studies retrieved in the database were identified using the VOS viewer™ software. To increase the level of refinement of the map for citation visualization, the minimum number of citations and publications was defined as 1, and 173 were presented. The research groups formed four clusters: red, yellow, green, and blue. The most expressive of the clusters with the most robust nodes were red, with authors Iglesias-Linares A, Solano Reina E, Mendoza-Mendoza A, Ortiz Ariza E, and Yañez-Vico RM highlighted. Other prominent names in the clusters included Flores-Mir C (yellow cluster), Hartsfield JK (green cluster), Macri JV (blue cluster), and Al-Qawasmi RA (blue cluster) (Fig 2). The size of the nodes in the visualization represents the impact of each researcher’s publications, measured by the number of citations received. The larger the node, the greater the author’s impact and relevance in the research field. The edges of the maps show scientific collaboration between the four clusters independently, where the red cluster is connected to the yellow and green clusters, the green cluster is connected to the red and blue clusters, and the blue and yellow clusters are not connected, showing that there is no co-authorship collaboration in this case.

### MAIN RESEARCH CENTERS

**Figure 3:**
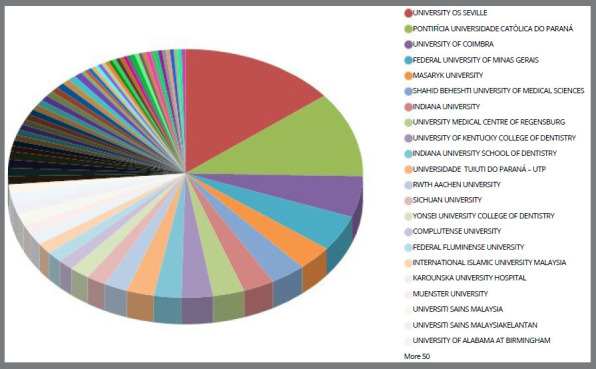
Most cited organization centers.

Research centers related to the topic were identified, with the five most relevant centers being: the University of Seville (Spain), Pontifical Catholic University of Paraná (Brazil), University of Coimbra (Portugal), Federal University of Minas Gerais (Brazil) and Masaryk University (Czech Republic) (Fig 3).

### MAIN COUNTRIES IN TERMS OF CITATION AND COLLABORATION

**Figure 4:**
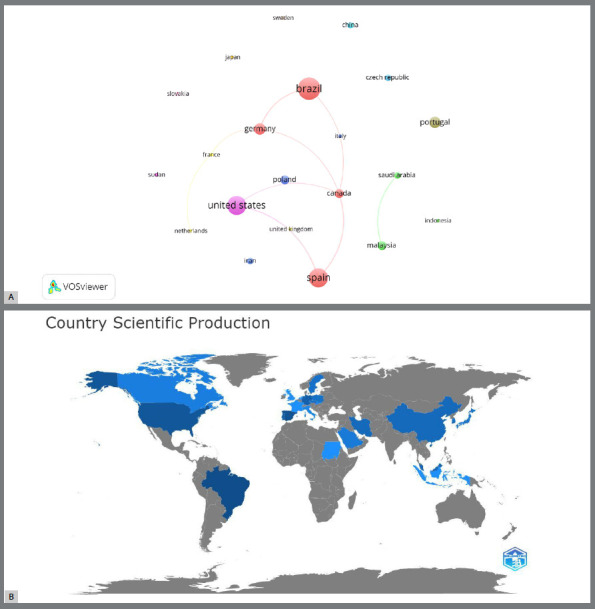
A) Network visualization map of top countries regarding citation. B) Global visualization map of the countries that search the most on the topic. The darker the shade of blue, the more intense the scientific collaboration within the theme, with the countries shown in navy blue being the most prominent in this area of research as well.

Regarding the geographical distribution of citations on the topic, when defined for 1 publication and 1 citation, 22 countries were found, as shown in Figure 4A. Brazil, the USA, Spain, and Germany surpassed with maximal nodes, and consequently, in research on this topic. The margins of collaboration between countries showed a significant link between Brazil and Germany, whereas the USA showed superior collaboration with Spain and Canada. Other independent clusters, including Portugal, China, Iran, the Czech Republic, Poland, South Korea, Sweden, Japan, Slovakia, Indonesia, and Sudan, appeared separately on the map. According to the map, Malaysia and Saudi Arabia cooperate (Fig 4B). To make viewing even easier, the world map created by the Bibliometrix software illustrates the countries that publish the most about the topic. The world map created by the bibliometric software illustrates the countries publishing the most on the topic, to facilitate visualization further. In this map, the darker the shade of blue, the more intense the scientific collaboration within the theme, with the countries shown in navy blue being the most prominent, consistent with the network map explained above. 

### ANALYSIS OF THE MAIN KEYWORDS

**Figure 5:**
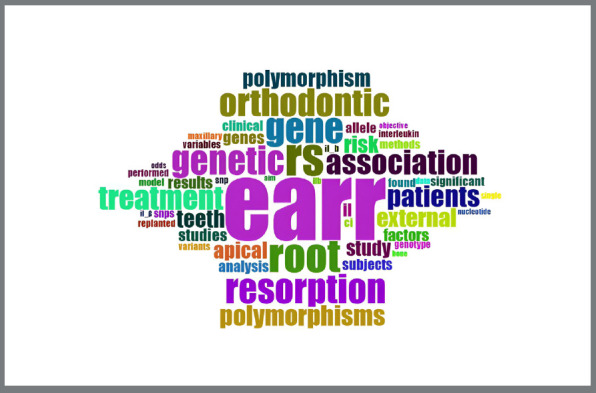
Word cloud for analysis of the co-occurrence of keywords in the abstract.

When analyzing the co-occurrence of keywords, we chose to analyze the keywords mentioned in the abstracts. Figure 5 enables the identification of a cloud of relevant words and acronyms. The use of the acronym EARR significantly increased the number of publications. In the field of genetics, the terms “polymorphism”, “rs”, and “gene” were frequent (Fig 5).

In the Figure 5, the acronym “EARR” is more prominent and larger, meaning that it appears a lot in this type of study, and it is crucial to use it if it makes sense for the author’s research. Words of the same color come close to the theme and have a strong connection between them, functioning as clusters, such as “SNPs” and “resorption” or “genetic”, “EARR” and “genotype”.

Some words must appear less expressively in the analyzed cloud of highlighted keywords, such as “IL-B” and “interleukin”, and this reveals interesting insights into the research area. Although less evident, interleukin was the only gene cited as a keyword in the abstracts, suggesting that it is one of the most studied genes within the topic covered. 

### MOST CITED SCIENTIFIC JOURNALS AND DOCUMENTS

In the Bibliometrix software, citation indicators are based on measuring the number of citations received by a given publication; therefore, the cutoff point supports the results referring to the impact and number of citations of the articles and becomes an evaluation parameter, by relating the number of scientific publications with the number of citations.^16^

**Figure 6:**
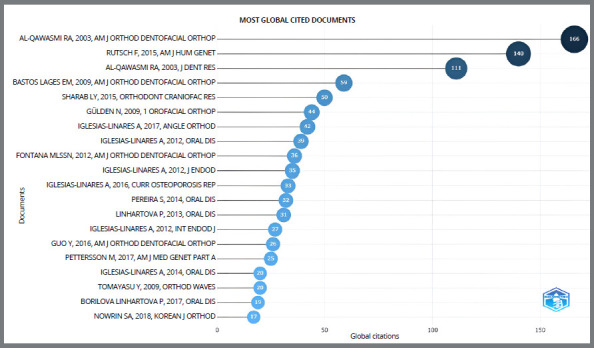
Global visualization of the most cited documents.

**Figure 7:**
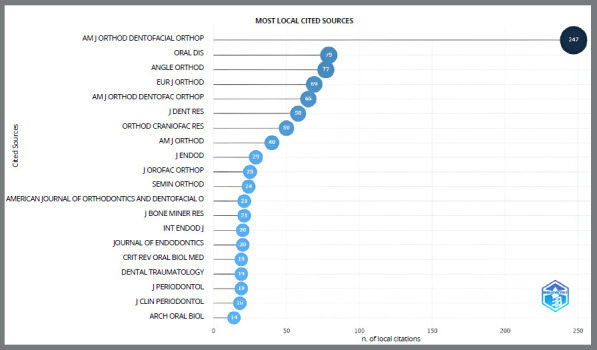
Global visualization of the most cited sources (journal).

A total of 20 documents were highlighted based on citation number (Fig 6). The 20 most-cited articles were published between 2003 and 2018. Among these, nine^2^ ^,^ ^5^ ^,^ ^7^ ^-^ ^9^ ^,^ ^12^ ^,^ ^17^ ^-^ ^19^ investigated the polymorphism in the IL gene polymorphisms as predictors of EARR. Of the 20 articles highlighted, 16 are from the area of orthodontics (15 studies and 1 systematic review) and 4 are from other areas of Dentistry, such as Endodontics. The number of citations varied between 17 and 166 citations per year. Among the most cited documents, the reference “Al-Qawasmi RA et al., 2003”^3^ led the ranking, with 166 citations. Figure 7 shows the “American Journal of Orthodontics and Dentofacial Orthopedics” (appears with 4 different abbreviations) as the most cited magazine.

### MOST RELEVANT AUTHORS

**Figure 8:**
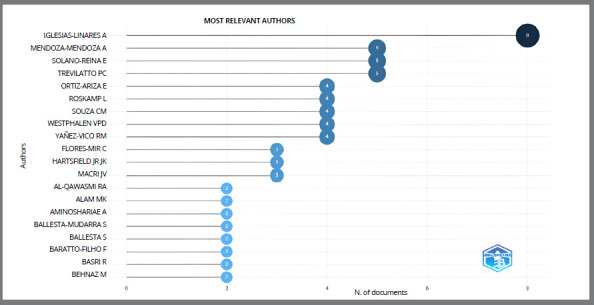
Global visualization of the most relevant authors.

**Figure 9:**
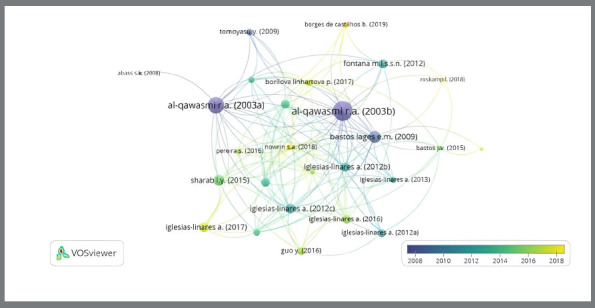
Global visualization of the most relevant authors and the timeline of corresponding publications.

The most relevant authors, including the main authors and also co-authors by number of citations for this research topic, are listed in Figure 8. The most relevant authors from the most-cited publications on this topic ranged from two to eight. The graph in Figure 9 shows the primary authors who published on the topic and the timeline of the corresponding publications. It is important to note that the timeline varied from 2008 to 2018, with an increase from 2014 onwards, with the green and yellow clusters standing out due to the number of interconnected nodes and edges. The more robust nodes in purple shades, featuring the author Al-Qawasmi, have a greater volume; this may indicate he was the reference author between 2003 and 2008, and his work probably functioned as a precursor to new research. Iglesias-Linares A, Mendoza-Mendoza A, Solano-Reina E, and Trevilatto PC prevail over others, for publishing much scientific literature in this specific area of knowledge.

Among the most cited articles were the first publications dated 2003,^5^ followed by stagnation of publications on the subject and subsequent considerable growth in the number of publications between 2009^6^ ^,^ ^17^ ^,^ ^28^ and 2018.^4^

### NARRATIVE DATA SYNTHESIS

Narrative data synthesis of the 44 included articles^2^ ^-^ ^10^ ^,^ ^12^ ^,^ ^17^ ^-^ ^50^ are presented on Tables 1, 2 and 3. Tables 1 and 2, which provide a narrative analysis, were based on genetics and EARR studies, the most cited on orthodontics, and Table 3 presents an analysis of the reviews on the topic included in the selection.

EARR severity was assessed in the upper and lower central incisors and mesial and distal roots of the lower first molars. The analysis indicated that the IL-1B polymorphism was responsible for 15% of the total variation in upper incisor EARR. Only one of the most cited studies investigated the TaqI polymorphism of the vitamin D receptor as a possible predisposing factor for EARR.^20^ It is important to highlight that genes such as SPP1, P2RX7, TNFRSF11B, TNFRSF11A,^17^ ^-^ ^19^ and the osteopontin gene cluster (rs9138 and rs11730582)^21^ were also highlights of the research among the most cited articles. Another study^20^ showed that different genetic polymorphisms may indicate the occurrence of EARR in individuals undergoing orthodontic treatment. The IL-1B polymorphism was not associated with an EARR predisposition, but heterogeneity among the study results was significant; however, this remains unexplained. Although IL-1 B is considered a promising gene for predicting EARR in patients undergoing orthodontic treatment, better-controlled studies are required to verify this association. People who are homozygous for IL-1B allele 1 have an increased risk of EARR, compared to those who are not homozygous for IL-1 beta allele 1. Defining the genetic contributions to EARR is important for understanding the contribution of environmental factors, such as habits and therapeutic biomechanics (Table 1).

Considering the importance of providing information that may be useful for daily clinical practice and for researchers in the field of Orthodontics, important variables were presented in Table 2, regarding the most cited orthodontic studies, such as: study design, study population, sample size, average age, EARR assessment methods, teeth selected for EARR assessment, orthodontic technique used and Angle classification.

Considering the reviews on the topic, the narrative reviews of the literature highlight a series of genes studied, placing great importance on the study of interleukins.^45^ ^,^ ^46^ ^,^ ^50^ The systematic reviews suggest guidelines and recommendations for future researchers who study genetic polymorphism in patients undergoing orthodontic treatment,^4^ ^,^ ^24^ and are not conclusive regarding the association of IL with EARR.^3^ ^,^ ^23^ In a meta-analysis, P2RX7 (rs208294) indicated the risk of orthodontic patients for EARR with a very low quality of evidence, according to GRADE^3^ (Table 3). It is important to highlight that the level of evidence on this topic has increased over the years, culminating in the first systematic review^4^ publication in 2018. A patterned increase in the number of manuscripts over the years ensures that there will also be an increase in the level of evidence presented.

## DISCUSSION

Dentistry research needs to be constantly updated for clinical practice. Metric studies such as bibliometric reviews allow the analysis of scientific production to obtain results that bring, in addition to quantitative data, possibilities for qualitative and representative analyses of various areas of knowledge.^51^ ^,^ ^52^

The practice of evidence-based Dentistry helps highlight the importance of each study design because there is an appropriate delineation capable of answering each clinical question. The main goal is to help clinicians perform more effective, efficient, and predictable treatments and make decisions based on robust scientific evidence and patient behavior. In this case, we noticed the tendency for publications of reviews, concise reviews, systematic reviews and meta-analyses on the topic.^3^ ^,^ ^4^ ^,^ ^23^ ^,^ ^24^ ^,^ ^45^ ^,^ ^46^ ^,^ ^50^ In this bibliometric analysis, we can observe its peculiarities and observe the significant increase in scientific evidence concerning the topic presented over the years. 

Before starting a systematic review, one should identify its real need, through the search for secondary studies with the same theme and objective. So a bibliometric analysis presents fundamental importance for to be able to evaluate a general panorama on the subject researched and to visualize the interaction of other studies of their interest, in addition to expanding the possibilities of access to other articles in the area.^52^

Furthermore, as EARR is unpredictable and depends on multiple factors, it is extremely important to carry out a careful and complete diagnosis through history and periapical radiographic examinations, so that rational mechanotherapy can be planned. This must be accessed based on literature, and bibliometrics can be a quantitative method for accessing existing scientific research.

### MAIN FINDINGS

Regarding collaboration between authors, organizations, and countries, the USA is dominant since most of the high-impact scientific journals are North American; however, the presence of Brazil also indicates that the intellectual production of Brazilian researchers has significant scope in the international scene.

The journals “American Journal of Orthodontics and Dentofacial Orthopedics”, “Oral Diseases”, “The Angle Orthodontist”, and “European Journal of Orthodontics” are of great relevance in the area, demonstrating a high number of publications on the subject and a great impact factor within the literature. Highly productive researchers such as Iglesias-Linares A, Flores-Mir C, Hartsfield J K, and Al-Qawasmi RA formed a group of reference authors. 

With scientific mapping, it is also possible to monitor temporal evolution and identify the main authors involved.^52^ In addition, the notable network of researchers over the years has been less concentrated on just one specific author, as in 2008 and 2009, when studies on polymorphisms and EARR were concentrated on Al-Qawasmi RA^5^ ^,^ ^27^ and Bastos Lages.^6^

In this bibliometric analysis, authors such as Iglesias-Linares A, Flores-Mir C, Hartsfield JK, and Al-Qawasmi RA formed a prominent group in the scientific literature on the subject. The first author to be highlighted is Iglesias-Linares A, and the map of the visualization network shows the author with a large node in the red cluster. However, it is possible to see that the clusters of authors are interconnected subtly, without showing great support or partnerships, suggesting that these studies are concentrated in countries in isolation. It would be interesting for these authors to form partnerships with other leading authors in the field, to cover the research and deepen our knowledge of genetic polymorphisms and EARR. The USA appears in an independent cluster and is very prominent in terms of the number of documents published and cited, but is less prominent in terms of recent impact, which suggests that more recent publications have not yet had enough time to have many citations. 

### GEOGRAPHICAL TRENDS

Brazil, the USA, Spain, and Germany are the most prominent countries, highlighted in navy blue on the map. South America and Africa have only been listed as prominent countries only once, which calls into question the conditions of technological advances in genetic studies on these continents. Europe is highly active in the field of genetic studies. Regarding the co-occurrence of keywords, the most prominent words referred to genetics and EARR, with interleukin to a lesser extent, which suggests that research has focused more on the study of these genes and, simultaneously, suggesting that this gene has already been extensively explored.

### STRENGTHS AND LIMITATIONS

The database chosen was Scopus, which is the largest database of peer-reviewed literature summaries and citations, with bibliometric tools to track, analyze, and visualize research. However, other databases exist that can be explored, and perhaps those may generate different results.

Furthermore, the number of articles found on the topic was minimal, which could generate research bias. However, it is a subject that is still negligibly explored in different specialties of Dentistry. In this way, bibliometrics has proven to be useful in scientific production in the fields of Genetics and Dentistry.

### RESEARCH GAPS AND POSSIBLE PROSPECTS FOR FUTURE RESEARCH

Most journals with more citations on this topic dealt with Orthodontics. Endodontics is an area of research that can focus more on root resorption and genetic polymorphisms, as they are topics that are still less explored, compared to Orthodontics.

One of the important points that can be observed in this study is the number of genetic studies focusing on interleukin polymorphisms. A recent systematic review and meta-analysis^3^ discovered, through qualitative analyses, that there are some genes involved in EARR susceptibility due to orthodontic tooth movement. The same study found, in the individual analysis of articles, that interleukins were more strongly associated with EARR; however, in the meta-analyses, by increasing the statistical power by grouping the studies, this finding was not confirmed. This result is consistent with a previous study by Nowrin et al.^4^, in which it was suggested that the IL-1B polymorphism is not associated with a predisposition to EARR in their meta-analysis. Therefore, the current evidence highlights a gap in the research, suggesting the need for larger multicenter studies to investigate new genes associated with EARR. 

## CONCLUSIONS

Based on the bibliometric analysis, it is concluded that there are still few research groups that explore this topic, with the studies being most explored in Spain, Brazil and the USA. Endodontics is an area of research that could focus more on root resorption and genetic polymorphisms, as it still underexplored compared to Orthodontics. Polymorphisms have been studied as possible predictors of EARR caused by orthodontic tooth movement. However, there is still a gap in research, indicating the need to search for new genes associated with EARR. 
